# Bioengineering of Tobacco Mosaic Virus to Create a Non-Infectious Positive Control for Ebola Diagnostic Assays

**DOI:** 10.1038/srep23803

**Published:** 2016-03-31

**Authors:** Patricia Lam, Neetu M. Gulati, Phoebe L. Stewart, Ruth A. Keri, Nicole F. Steinmetz

**Affiliations:** 1 Department of Biomedical Engineering, Case Western Reserve University, Cleveland, 44106, USA; 2 Department of Pharmacology, Case Western Reserve University, Cleveland, 44106, USA; 3 Cleveland Center for Membrane and Structural Biology, Case Western Reserve University, Cleveland, 44106, USA; 4 Department of Genetics, Division of General Medical Sciences-Oncology, Case Western Reserve University, Cleveland, 44106, USA; 5 Case Comprehensive Cancer Center, Case Western Reserve University, Cleveland, 44106, USA; 6 Department of Radiology, Case Western Reserve University, Cleveland, 44106, USA; 7 Department of Materials Science and Engineering, Case Western Reserve University, Cleveland, 44106, USA; 8 Department of Macromolecular Science and Engineering, Case Western Reserve University, Cleveland, 44106, USA

## Abstract

The 2014 Ebola epidemic is the largest to date. There is no cure or treatment for this deadly disease; therefore there is an urgent need to develop new diagnostics to accurately detect Ebola. Current RT-PCR assays lack sensitive and reliable positive controls. To address this critical need, we devised a bio-inspired positive control for use in RT-PCR diagnostics: we encapsulated scrambled Ebola RNA sequences inside of tobacco mosaic virus to create a biomimicry that is non-infectious, but stable, and could therefore serve as a positive control in Ebola diagnostic assays. Here, we report the bioengineering and validation of this probe.

The 2014 Ebola outbreak in Africa is the largest Ebola epidemic to date, with approximately 28,000 reported cases worldwide, with the majority in West Africa, and isolated cases in the United States and parts of Europe^1^. The possible spread of the Ebola virus (EBOV) from West Africa to other parts of the world has been a cause for great concern due to the highly pathogenic and deadly nature of Ebola.

Presently, there are no approved vaccines and treatments of EBOV infection, though there are many in research and development^2^. The most promising vaccines are recombinant virus-like particles that express EBOV glycoproteins^3^. For example, an adenovirus-based vaccine (ChAd3-ZEBOV) is in Phase 1 trails^4^,^5^,^6^, and a recombinant vesicular stomatitis virus (rVSV)-vectored vaccine (rVSV-ZEBOV) entered clinical trials in April 2014 and is currently in Phase 3 evaluation^7^,^8^,^9^,^10^,^11^. The rVSV-ZEBOV vaccine has been shown to protect vaccinated macaques from lethal doses of the EBOV, providing evidence that it may work in humans^12^. While several small molecule approaches, including antivirals and RNA-based silencing approaches, are undergoing development and testing for treatment of the disease^13^,^14^, the most promising therapeutic strategy to date is ZMapp, a cocktail containing three chimeric monoclonal antibodies. ZMapp was experimentally used to treat patients during the 2014 outbreak in West Africa, although its efficacy has not yet been evaluated in clinical trials^15^.

Though much progress has been made in developing vaccines and treatments, detection and monitoring is the most effective, and currently the only option to prevent the spread of the disease^16^. Early symptoms of EBOV infection are non-specific and may include fever, headache, unexplained bleeding, fatigue and vomiting. Since the symptoms of EBOV infection cannot generate an accurate diagnosis, reliable diagnostic tests are required. Current diagnostic assays approved by the Food and Drug Administration (FDA) assess the presence of EBOV-specific antigens using immunoassays, or EBOV genome-specific sequences detected by reverse transcription quantitative real-time polymerase chain reaction (RT-qPCR)^17^. Immunoassays screen blood and/or serum samples from suspected patients for Ebola-specific antibodies, and can rapidly provide results when laboratory facilities are not available. RT-qPCR-based assays target the nucleic acid^18^,^19^, and require sophisticated laboratory equipment and facilities. While immunoassays, such as the ReEBOV Antigen Rapid Test kit developed by Corgenix, accurately detect EBOV, PCR-based assays are more sensitive as they can detect the presence of EBOV up to 72 hours prior to antigen detection^18^. However, the reliability of RT-qPCR tests is dependent on the availability of accurate positive controls to circumvent false negative results. Such internal, positive controls are essential for confirming the performance of the assays and negate the potential presence of processing errors that pre-empt the enzymatic RT-PCR steps.

RT-PCR tests such as the EZ1 Real-time RT-PCR Assay designed by the Department of Defense, or the Center for Disease Control and Prevention (CDC)’s Ebola Virus Real-time RT-PCR Assay, use synthetic RNA transcripts as a positive control in the assay. However, these ‘naked’ RNAs are unstable molecules that are highly susceptible to degradation. The synthetic RNAs must be stored in a freezer, and only survive limited freeze/thaw cycles^20^. Due to the inherent instability of these molecules, they cannot be used to spike samples during the initial sample processing steps that are the most likely to be impacted by handling errors. Rather, these synthetic control RNAs are only used during the reverse transcription and amplification steps of the assay. An improvement is the FilmArray assay by BioFire Defense, which uses the yeast *Schizosaccharomyces pombe* to encapsulate the control RNA to protect it. This positive control is added to samples at the initial stage of sample preparation with the goal of demonstrating that all steps of the assay from sample extraction to PCR have been successfully completed. When the FilmArray assays were compared to traditional RT-qPCR assay, it was shown that there were eight discordant results out of 60 samples, indicating that the FilmArray assays are an effective alternative to RT-qPCR^21^. However, a limitation of this approach is that yeast does not mimic the structure and stability of the viral nucleoprotein complex present in EBOV.

To overcome the issue of the lack of a sensitive and reliable internal control in Ebola diagnostics, we set out to create a biomimicry of EBOV that could be added to samples prior to any preparatory steps. Specifically, we used a plant virus, the tobacco mosaic virus (TMV) that mimics the structural features of EBOV. In this work, we made use of these RNA-programmed self-assembly properties to design and engineer a TMV-based probe containing scrambled EBOV sequences for its use as a positive control in Ebola diagnostics.

## Results

### Design and Synthesis of the Ebola-TMV mimic

EBOV is a filamentous virus belonging to the *Filoviridae* family. The EBOV genome is comprised of a single-stranded RNA molecule packaged into 7 proteins: the nucleoprotein (NP), viral proteins (VP) 24, 30, 35 and 40, glycoprotein (GP) and an RNA-dependent RNA polymerase (L). The nucleoprotein complex is encapsulated in a lipid envelope that is embedded with glycoproteins^22^. TMV is a filamentous single-stranded RNA virus, which, like EBOV, forms a nucleoprotein complex to protect its single-stranded RNA genome; therefore TMV mimics EBOV much more closely than yeast. The structure of TMV is well characterized: TMV consists of 2,130 identical copies of a 17.5-kDa coat protein that self-assemble into a 300 × 18 nm filament encapsulating the single-stranded RNA genome. The self-assembly of TMV is well understood^23^,^24^,^25^,^26^; the particles can be disassembled and reassembled using synthetic RNA transcripts containing the origin of assembly site (OAS), a 234 nucleotide (nt)-long sequence that initiates nucleoprotein assembly. Through bioengineering design, synthetic RNAs of various length and sequence can be encapsulated into TMV coat proteins forming TMV rods of defined aspect ratio, or even complex structures, such as star- or boomerang-shaped assemblies^27^,^28^,^29^,^30^,^31^.

**Figure 1:**
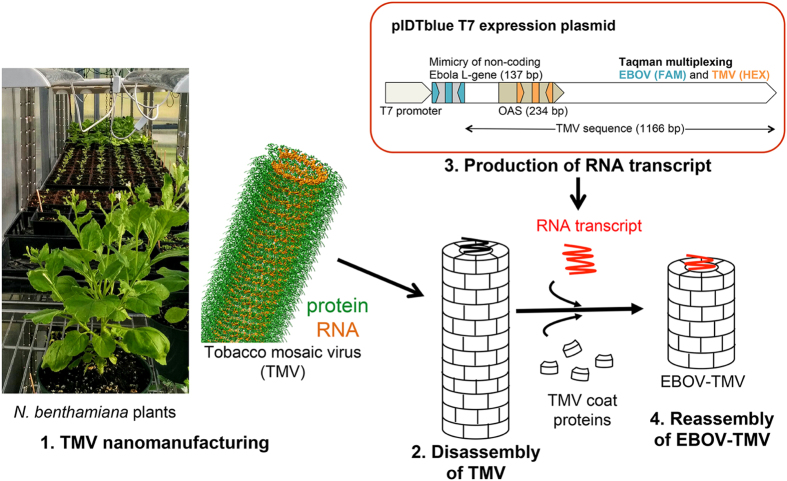
Workflow used to nanomanufacture EBOV-TMV nanoparticles. From left to right: *Nicotiana benthamiana* plants can be used to produce TMV particles. The structure of TMV is shown highlighting the protein in green and its RNA in orange (the image was created using 2TMV PDB file and the UCSF chimera software [http://www.cgl.ucsf.edu/chimera]). Extracted TMV is dissembled followed by reassembly using purified TMV coat proteins (CPs) and synthetic RNA transcripts to yield the Ebola-TMV mimic (EBOV-TMV). A schematic of the RNA transcript design is shown above (for detailed information see Supplementary Information).

The workflow that was used to nanomanufacture the Ebola-TMV mimic (EBOV-TMV) nanoparticles is shown schematically in Fig. 1. TMV particles were produced using *Nicotiana benthamiana* plants. Infection was induced through mechanical inoculation and leaves were harvested 2 weeks post-infection; TMV was extracted using chloroform:butanol extraction and PEG precipitation as we previously described^32^. The particles were then disassembled and the genome removed using the acetic-acid-method described by Fraenkel-Conrat^33^. Purified coat proteins (CPs) were then reassembled using a synthetic RNA transcript containing the TMV OAS as well as EBOV specific sequences. The specific portions of the EBOV RNA-dependent RNA polymerase gene (L-gene) were selected in regions that showed sequence homology between all the published EBOV sequences (Supplementary Information, Fig. S1a)^34^,^35^. To generate the template RNA, a gene fragment of 160 base pairs (bp) was synthesized containing a 137 bp segment of the EBOV L-gene flanked with restriction enzyme sites (Supplementary Information, Fig. S1b). This gene fragment was cloned in a pIDTblue T7 expression vector upstream of a shortened TMV sequence 1166 bp long (the full length TMV genome is 6,395 nt in length) containing the 234 bp-long OAS to generate the plasmid pIDTblue/EBOVL1-TMVshort (Supplementary Information, Fig. S2). This plasmid was then produced in *Escherichia coli*, isolated, and then used as a template for *in vitro* transcription of the synthetic EBOV-TMV. Synthesized EBOV-TMV RNA was then incubated with purified CPs at 30 °C for 16–20 hours to assemble EBOV-TMV nanoparticles. The EBOV-TMV assembly was purified by ultracentrifugation over a 40% sucrose cushion to remove any excess, free RNA or CPs from the mixture.

### Characterisation of EBOV-TMV particle

UV/visible spectroscopy was used to determine the concentration and yield of synthesized EBOV-TMV. The concentration was determined using the TMV-specific extinction coefficient of 3.0 cm^−1^ mg^−1^ ml (at 260 nm); EBOV-TMV was produced at yields of 0.34 mg from a self-assembly reaction containing 0.52 mg CPs and 20 μg RNA transcripts (65% yield). Further, the A260/A280 ratio of the EBOV-TMV particles was measured at 1.12, which is indicative of intact particles (1.1 ± 0.1)^36^.

**Figure 2:**
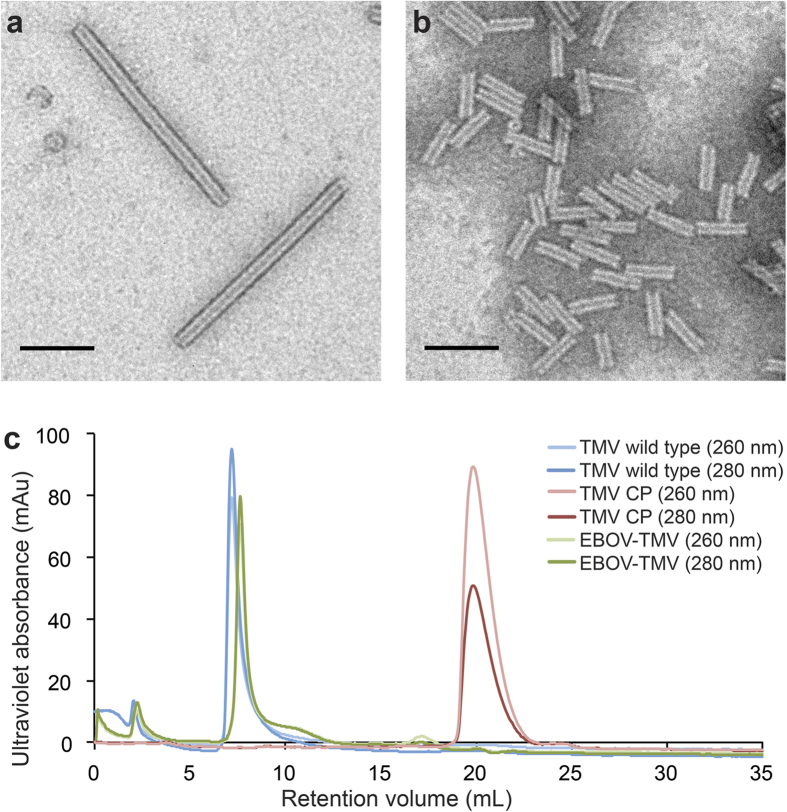
Characterisation of TMV and reconstituted EBOV-TMV. Transmission electron micrographs of negatively-stained (**a**) wild-type TMV rods, (**b**) EBOV-TMV rods (scale bar = 100 nm). (**c**) Size exclusion elution profiles using a Superose6 column and Äkta Purifier of TMV wild type, TMV coat proteins (CP) and EBOV-TMV nanoparticles; the detectors were set at 260 and 280 nm: light blue/red/green = 260 nm, dark blue/red/green = 280 nm.

To confirm the structural integrity of the EBOV-TMV constructs, we performed transmission electron microscopy (TEM) using negatively stained samples (Fig. 2a,b). Compared to the wild-type TMV rods that are 300 nm in length, the EBOV-TMV appeared, as expected, much shorter. ImageJ^37^ was used to measure the lengths of the rods from multiple TEM micrographs. 422 EBOV-TMV rods were analysed (Table 1). On average, the rods measured 61.3 ± 8.8 nm in length, which is in agreement with the predicted length (based on the length of the synthetic RNA transcript). The size of the assembly was also confirmed by dynamic light scattering (DLS) (Table 2). While the effective diameter measurements only provide an approximation for the high aspect ratio materials, the DLS measurements were in good agreement with the TEM studies and also indicated that the EBOV-TMV preparations are monodisperse. The long-term stability of the particles was also assessed by TEM. After two months of storage at 4 °C, the assembled particles remained intact (Supplementary Information, Fig. S3).

TMV, free CPs, and EBOV-TMV formulations were then analysed by size-exclusion fast protein liquid chromatography (FPLC) (Fig. 2c). TMV showed the typical elution profile, due to the large size (300 × 18 nm) and high molecular weight (40 MDa), the particles are eluted at the void volume of the column (7–8 mL); free CPs elute at 21 mL. The EBOV-TMV elution profile matched the TMV profile; the absence of the 21 mL-peak confirms that the EBOV-TMV preparation was free of non-assembled CPs or RNA contaminants. The slight delay of the EBOV-TMV elution compared to TMV (7.6 mL vs. 7.1 mL) may reflect the smaller size of the EBOV-TMV samples (60 × 18 nm, 8 MDa).

### Validation of the EBOV-TMV probe with RT-qPCR

Primers and hydrolysis probes (Taqman probes) were designed to target the EBOV L-gene sequence as well as the TMV OAS (Supplementary Information, Fig. S1, Table S1). The EBOV L-gene probe was labelled at the 5′ end with fluorescein (FAM) whereas the TMV OAS probe was 5′ labelled with hexachlorofluorescein (HEX). Both probes were quenched with the Iowa Black Fluorescence Quencher attached to the 3′ end. First, linearized plasmid DNA (pIDTblue/EBOVL1-TMVshort) was used to optimize the reaction conditions for the qPCR assay (Supplementary Information, Fig. S4). Once the reaction was optimised, EBOV mimicries were studied.

**Figure 3:**
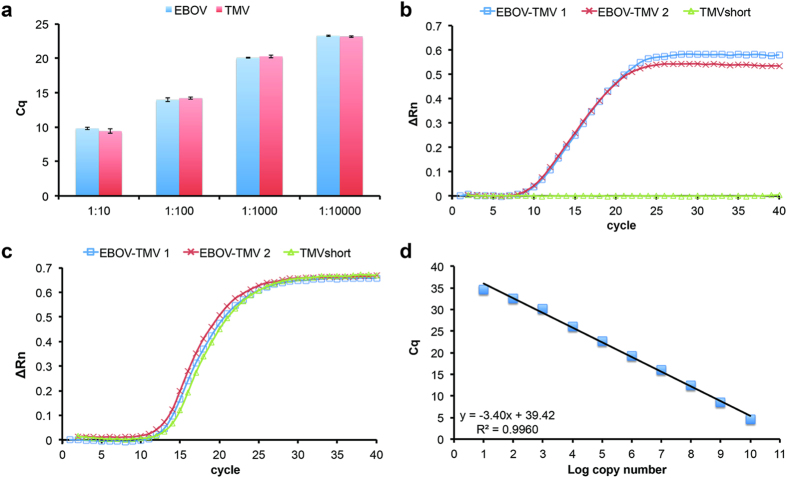
Validation of the qPCR assay and EBOV-TMV control sample. (**a**) RNA was extracted from spiked-in EBOV-TMV probe and converted to cDNA. qPCR of serial dilutions of cDNA detected EBOV and TMV at similar quantification cycles (Cq). (**b**) One-step RT-qPCR to detect for the presence of EBOV from independent samples (threshold = 0.026). (**c**) One-step RT-qPCR to detect for the presence of TMV from independent samples (threshold = 0.066). (**d**) Limit of detection of the one-step RT-qPCR assay. Serial dilutions of 10^10^ copies to 10^0^ copies were assayed to determine the limit of detection for EBOV.

To validate the functionality of the EBOV-TMV probe for Ebola diagnostics, we performed qPCR and RT-qPCR assays. First, RNA was extracted from EBOV-TMV particles, followed by synthesis of first-strand cDNA by reverse transcription using a mixture of random primers. Serial 10-fold dilutions of cDNA were assayed to validate detection of the EBOV-specific RNA sequence. As a proof-of-concept, a multiplexed assay was performed to detect both the EBOV and TMV OAS. A dilution series (1:10^2^ to 1:10^4^) confirmed detection of both EBOV and TMV RNA in the same quantification cycle (Cq) as expected (Fig. 3a).

Next, one step RT-qPCR was performed. RNA was extracted from EBOV-TMV particles spiked in phosphate-buffered saline (PBS) followed by one-step RT-qPCR. EBOV-TMV as well as short TMV nanoparticles (TMVshort) lacking the EBOV-specific sequences were considered. TMVshort particles were assembled using a 1166 nt RNA transcript that lacked the EBOV-specific sequences and served as a negative control in the assay. Using the EBOV-TMV sample, both EBOV (Fig. 3b) and TMV (Fig. 3c) were detected; the TMVshort negative control proved negative for EBOV and positive for TMV, as expected (Fig. 3b,c). These results attest to the specificity of the developed assay.

Lastly, the sensitivity (limit of detection) of the assay was evaluated using 10-fold serial dilutions (10^10^ to 10^0^ copies) of RNA in a one step RT-qPCR reaction. The limit of detection was defined as the concentration where 95% of the samples result in positive amplification. For EBOV, amplification could be detected from 10^10^ (mean Cq = 4.60) to 10^1^ copies (mean Cq = 34.69). No amplification was detected in all 4 replicates of 10^0^ copies. When the log copy numbers were plotted against mean Cq, the efficiency was 96.73% and the correlation coefficient, R^2^, was 0.99 (Fig. 3d).

## Discussion

We have developed a novel positive control for use in Ebola diagnostic assays: a bioengineered TMV encapsulating RNA sequences with EBOV specificity. Because the nucleoprotein assembly realistically mimics the stability of the EBOV, the EBOV-TMV probe could be spiked into patient samples prior to processing and therefore function as an internal standard. As an alternative, the EBOV-TMV could be processed in parallel with clinical samples to ensure that all steps are performed correctly. The availability of a realistic positive control is critical to negate false negative results, especially when dealing with such a deadly disease as Ebola.

The TMV-based EBOV mimic is non-hazardous; the intervening sequences between the EBOV primer binding sites were scrambled so that the synthetic EBOV sequences do not translate into or encode self-replicating or infectious sequences. Further, the designed probe contains a shortened TMV sequence that carries only the 3′ end of TMV, partially encoding the CP, but lacks its movement protein and RNA-dependent RNA polymerase, and therefore also does not present an agricultural hazard. The shorter probe is also advantageous for bioengineering and scale up because shorter sequences result in higher yields of RNA transcripts. Supporting its use in remote locations, the EBOV-TMV probe can be stored for extended periods at 4 °C, and even room temperature without special handling.

From a nanomanufacturing point of view, the entire production process is economic and scalable; farming of infected tobacco plants typically yields 1–5 grams of TMV per kilogram of infected leaves and *E. coli* cultures can be scaled up to produce up to 1.5 milligrams of plasmid DNA per litre of bacterial culture. 3–5 mg RNA can be obtained per 0.1–1 μg DNA starting material through *in vitro* transcription. Overall, the nanomanufacturing of the probe follows a straightforward protocol that is scalable and could be translated to encapsulate a variety of sequences, not limited to infectious disease targets.

## Methods

### Alignment of the Ebola Genomes

Zaire Ebola genomes (GenBank accession numbers NC_002549.1 and KM034562.1) were aligned using Seqman (DNAstar). NC_002549.1 is the reference genome originally deposited at the NCBI. KM034562.1 is derived from sequencing the strain responsible for the 2014 outbreak in Africa, which discovered many new polymorphisms in the Ebola genome^35^. Consensus regions in the L-gene between the two genomes were identified. Primers for RT-qPCR were designed only in regions that showed homology.

### Cloning of pIDTblue/EBOVL1-TMVshort

Gene fragments for EBOVL-1 and TMVshort were synthesized by Integrated DNA Technologies (IDT). pIDTblue/TMVshort was generated using 1166 nt of the TMV genome (Accession NC_001367.1, positions 5374-6246) with *Hin*dIII and *Bam*HI restriction enzyme sites added to the 5′ and 3′ ends, respectively. A 137 nt region of the EBOV L-gene (Accession NC_002549.1, positions 12528-12664) was selected that harbours the primer binding sites (therein referred to as EBOVL-1). The regions in between the primer binding sites were scrambled with random TMV sequences so that the EBOV fragment would not be hazardous and pathogenic. This fragment was synthesized with 5′ *Eag*I and 3′ *Hin*dIII restriction enzyme sites. EBOVL-1 was cloned upstream of the TMVshort sequence into pIDTblue/TMVshort using the restriction enzyme sites 5′ *Eag*I and 3′ *Hin*dIII. Sanger sequencing was used to confirm the fidelity of the sequences.

### Template RNA Synthesis

The plasmids pIDTblue/EBOVL1-TMVshort and pIDTblue/TMVshort were linearized using *Bam*HI and purified by precipitation with 3M sodium acetate followed by Proteinase K treatment and phenol:chloroform extraction. Linearized plasmid was quantified on a Nanodrop2000 (Thermo Fisher). 1 μg of linearized plasmid was used for *in vitro* transcription using the MEGAscript T7 Transcription Kit (Ambion) as per manufacturer’s protocol. Following *in vitro* transcription, the RNA was purified by the MEGAclear Transcription Clean-Up Kit (Ambion) and stored at −80 °C until required.

### Plant growth and TMV propagation

Infection of TMV in 4–6 week old *Nicotiana benthamiana* plants was induced through mechanical inoculation using 100 ng/μL purified TMV in 0.1M potassium phosphate buffer, pH 7.0. Plants were propagated for 2–3 weeks post inoculation and TMV was purified according to Bruckman *et al*.^32^.

### Coat Protein Preparation

10 mg of purified TMV was treated with 2 volumes of glacial acetic acid for 20 minutes on ice. The sample was then centrifuged at 20,000 g at 4 °C for 20 minutes. The supernatant was removed and transferred to 6–8 MWCO dialysis tubing (Spectra/Por) and dialysed against ddH_2_O for 48 hours at 4 °C with one water change after 24 hours. Following dialysis, the coat proteins (CPs) were centrifuged at 20,000 × g at 4 °C for 20 minutes. The CPs were then resuspended in 75 mM sodium phosphate buffer (pH 7.2) overnight with gentle shaking. Absorbance at A250, A260 and A280 was measured on a Nanopdrop2000 (Thermo Fisher) to determine the integrity of the CPs. The concentration of the TMV CP was determined at A260 and ε = 1.3 μl μg^−1^ cm^−1^.

### Self-assembly of TMV Nanoparticles

Coat protein preparations and synthesized RNA transcripts were combined at a final concentration of 1.3 μg/μL and 50 ng/μL, respectively, in 75 mM sodium phosphate buffer (pH 7.2). The assembly reaction was incubated at 30 °C for 16–20 hours.

### Electron Microscopy

TMV and EBOV-TMV were diluted to 0.1 mg/mL in water and then adsorbed to Formvar- carbon-coated 400 mesh copper grids (Electron Microscopy Sciences) for 5 minutes, then washed with 20 μL ddH_2_O. Grids were then placed on a 20 μL drop of 2% (w/v) uranyl acetate for 5 minutes. Excess uranyl acetate was removed by blotting on filter paper before being imaged on a FEI Tecnai G2 Spirit transmission electron microscope at 100 kV.

### Size Exclusion Fast Protein Liquid Chromatography

Coat proteins and assembled particles were analysed by FPLC using a Superose6 column on the Äkta Purifier chromatography system (GE Healthcare). 100 μL of 1 μg/μL sample were analysed at a flow rate of 0.3 mL/min using 0.1M potassium phosphate buffer (pH 7.0). Detectors were set at 260 nm and 280 nm.

### RNA Extraction and Quantitative Reverse Transcription PCR

RNA was extracted from nanoparticles using TRI-Reagent (Sigma-Aldrich) as per manufacturer’s protocol. 100 ng of isolated RNA was used to generate cDNA using SuperScript VILO Master Mix (Thermo Fisher). For two-step RT-qPCR, cDNA was serially diluted and 2 μL was used in a reaction containing a final concentration of 1x DreamTaq PCR mix (Thermo Fisher), 300 nM of forward primer, 300 nM of reverse primer, 100 nM of probe. Reaction conditions were 95 °C for 2 minutes, followed by 40 cycles of 95 °C for 15 seconds, 60 °C for 1 minute. For one-step RT-qPCR, the SuperScript III Platinum One-Step Quantitative RT-PCR System with ROX (Thermo Fisher) was used. Synthetic RNAs for limit of detection assays were generated by *in vitro* RNA transcription of the pIDTblue/EBOVL1-TMVshort plasmid using the T7 Megascript kit (Ambion) and purified using the MegaClear kit (Ambion). Synthetic RNA was quantified using a Nanopdrop2000 (Thermo Fisher). Copy numbers per μL were calculated based on concentration and molecular weight of the synthetic RNA. 2 μL of RNA was used in a reaction containing a final concentration of 1x Reaction Mix with ROX, 300 nM of forward primer, 300 nM of reverse primer, 100 nM of probe and 0.4 μL of SuperScript III RT/Platinum Taq Mix. Reaction conditions were as followed: 50 °C for 15 min (for cDNA synthesis), 95 °C for 2 minutes, followed by 40 cycles of 95 °C for 15 seconds, 60 °C for 30 seconds. RT-qPCR was performed on a StepOnePlus Real Time PCR system (Thermo Fisher). All samples were run in quadruplicate. Quantification cycle (Cq) values were calculated for each reaction and data were analysed with the StepOne Plus software.

## Additional Information

**How to cite this article**: Lam, P. *et al*. Bioengineering of Tobacco Mosaic Virus to Create a Non-Infectious Positive Control for Ebola Diagnostic Assays. *Sci. Rep*. **6**, 23803; doi: 10.1038/srep23803 (2016).

## Supplementary Material

Supplementary Information

## Figures and Tables

**Table 1:** Length measurements of EBOV-TMV rods based on TEM imaging.

micrograph	length (nm ± SD)	n
1	64.11 ± 7.18	102
2	62.29 ± 8.93	110
3	59.35 ± 9.02	210
average	**61.27** ± **8.80**	**422**

422 EBOV-TMV particles from 3 different TEM micrographs were analysed using ImageJ. The predicted length of the rods based on the 1166 nt-long RNA template is approximately 60 nm. (SD, standard deviation; n, number of EBOV-TMV).

**Table 2:** DLS measurements of wild-type TMV and EBOV-TMV rods encapsulated with Ebola virus sequences.

**sample**	**effective diameter (nm ± SD)**	**polydispersity ± SD**
TMV wild-type	241.2 ± 2.3	0.204 ± 0.015
EBOV-TMV	69.8 ± 2.9	0.497 ± 0.044

Values represent mean ± standard deviation (SD); n = 5.
