# Supplementary material for: Bioengineering of Tobacco Mosaic Virus to Create a Non-Infectious Positive Control for Ebola Diagnostic Assays
Source: Sci Rep. 2016 Mar 31;6:23803. doi: 10.1038/srep23803 (PMC4814824; doi:10.1038/srep23803)
Supplement: Supplementary Information [file srep23803-s1.pdf]

Supplementary Information for

Bioengineering of Tobacco Mosaic Virus to Create a Non-Infectious Positive Control for Ebola Diagnostic Assays

Patricia Lam[1], Neetu M. Gulati[2,3], Phoebe L. Stewart[2,3], Ruth A. Keri[2,4,5] and Nicole F. Steinmetz[1,5,6,7,8,*]

[1]Department of Biomedical Engineering, Case Western Reserve University, Cleveland, 44106, USA
[2]Department of Pharmacology, Case Western Reserve University, Cleveland, 44106, USA
[3]Cleveland Center for Membrane and Structural Biology, Case Western Reserve University, Cleveland, 44106, USA
[4]Department of Genetics, Division of General Medical Sciences-Oncology, Case Western Reserve University, Cleveland, 44106, USA
[5]Case Comprehensive Cancer Center, Case Western Reserve University, Cleveland, 44106, USA
[6]Department of Radiology, Case Western Reserve University, Cleveland, 44106, USA
[7]Department of Materials Science and Engineering, Case Western Reserve University, Cleveland, 44106, USA
[8]Department of Macromolecular Science and Engineering, Case Western Reserve University, Cleveland, 44106, USA

*corresponding author: nicole.steinmetz@case.edu

a GGGCCGATTCTTAACACAAATGCATTTAGCTGTAAATCACACCCTRGAAGAAATTAC
AGAAATRCGTGCACTAAAGCCTTCACAGGCTCAMAAGATCCGTGAATTCCATAGAAC
ATTGATAAGGCTGGAGATGACGC

b tatGAGCTCCGGCCGttGGGCCGATTCTTAACACAAATGgttccaaagttgataaaa
taatggttcatgagaatgagtcattgTGCACTAAAGCCTTCACAGGCTCAtcagagg
tgaaccttcttaaaggagttGATAAGGCTGGAGATGACGCAAGCTT

Figure S1. Sequences of the Ebola RNA-dependent RNA polymerase (L-gene) used to create the synthetic RNA template for the EBOV-TMV nanoparticle. (a) The consensus sequence of the Ebola L-gene was surveyed using PrimerQuest (Integrated DNA Technologies) to identify suitable primer and probe binding sites for RT-qPCR. A 137 nt portion of the L-gene was selected (position 12528-12664), with the primers highlighted in yellow, probe in aqua. (b) The EBOV sequence was modified so that the intervening regions that do not correspond to primer and probe binding sites were scrambled (as represented by lowercase letters) so that the synthetic EBOV sequence did not translate into or encode self-replicating or infectious sequences. Restriction enzymes sites were added to facilitate cloning of the scrambled EBOV fragment (shown in red).

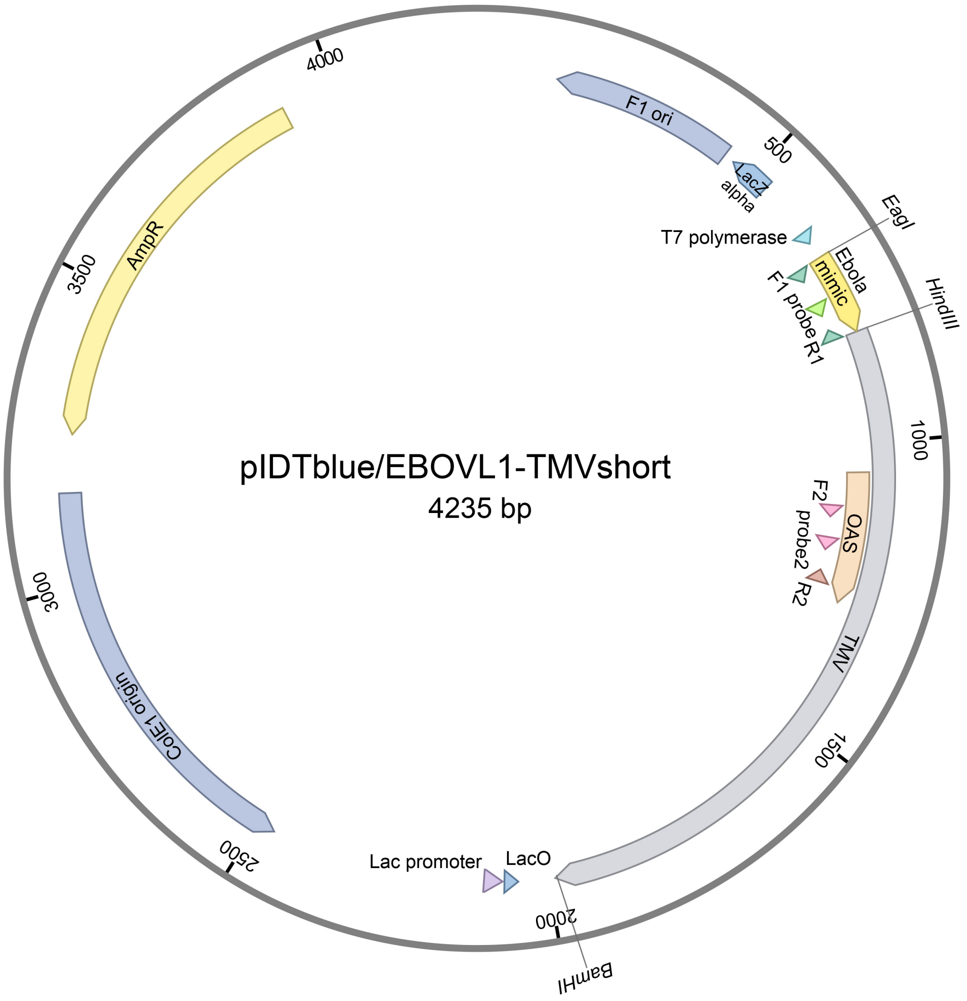

Figure S2. Genetic map of pIDTblue/EBOVL1-TMVshort. Primer and probe binding sites for RT-qPCR are indicated by triangles below the region they target. Genetic elements and restriction enzyme sites are also indicated: ori, origin of replication; lac, lactose; TMV, tobacco mosaic virus; OAS, origin of assembly; AmpR, ampicillin resistance site.

Table S1. Primers used for RT-qPCR.

EBOVL-1 – used to detect Ebola
Amplicon length: 137 bp

	Length	Tm	Start	Stop
Forward<br>5′-GGGCCGATTCTTAACACAAATG-3′	22	62.00	12528	12549
Probe<br>5′-TGCACTAAAGCCTTCACAGGCTCA-3′	24	68.00	12594	12617
Reverse<br>5′-GCGTCATCTCCAGCCTTATC-3′	20	62.00	12664	12645

TMV OAS2 – used to detect TMV
Amplicon length: 147 bp

	Length	Tm	Start	Stop
Forward<br>5′-GAGTTTGTGTCGGTGTGTATTG-3′	22	61.56	5374	5395
Probe<br>5′-TTACAAACGTGAGAGACGGAGGGC-3′	24	67.63	5435	5458
Reverse<br>5′-CCTGATTGACATAGGGACATCTT-3′	23	61.87	5520	5498

Probes were labeled with a reporter dye at the 5′ end (fluorescein (FAM) for Ebola and hexachlorofluorescein (HEX) for TMV), and Iowa Black Fluorescence Quencher as the quenching dye at the 3′ end.

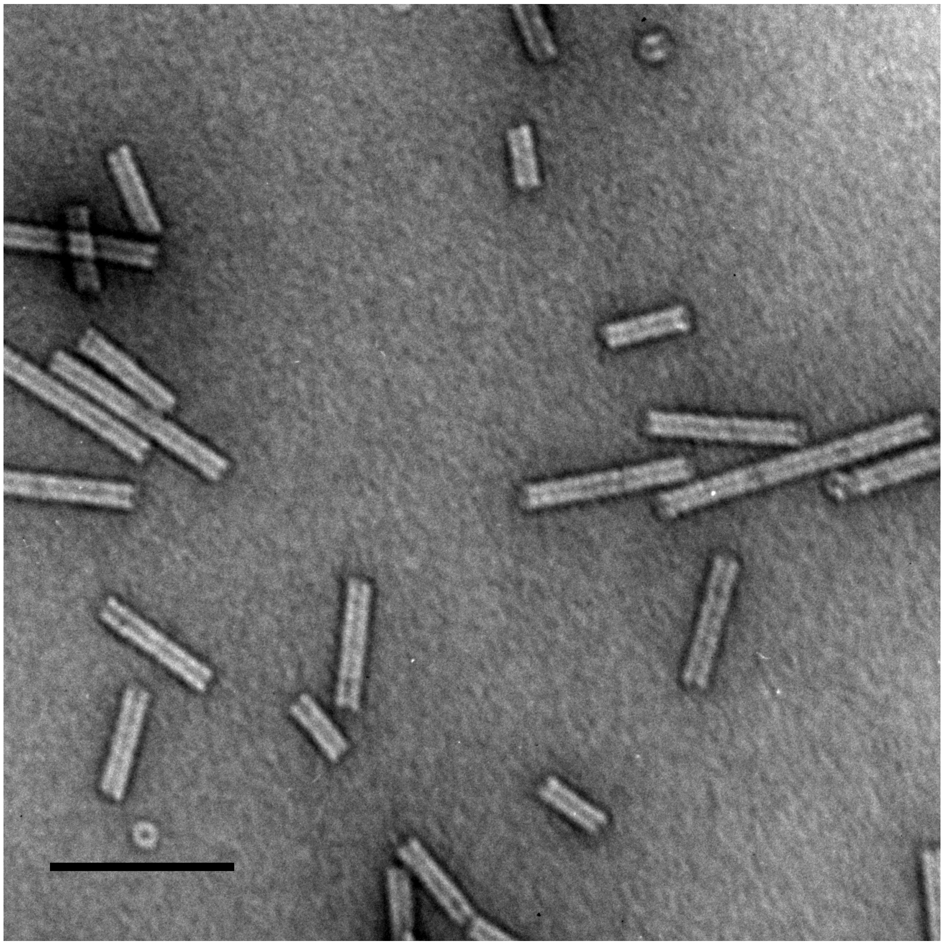

Figure S3. Assembled particles were stored at 4° C for 2 months, and re-imaged using transmission electron microscopy to verify the stability of the particles. After 2 months, the assembled particles remained intact. (scale bar = 100 nm)

Results and Discussion:

Optimization of qPCR conditions

A final primer concentration of 300 nM and a final hydrolysis probe concentration of 100 nM yielded the lowest minimum quantification cycle (Cq) without a decrease in fluorescence emission.

The sensitivity (limit of detection) of the assay was evaluated using quantified amounts of linearized pIDTblue/EBOVL1-TMVshort plasmid. 10-fold serial dilutions of 10^{10} copies/µL to 10^{1} copies/µL were tested using the same primer and probe sets designed for the diagnostic assay. For detection of the Ebola virus, the detection was linear over eight $\log_{10}$ steps from 10^{9} copies/µL (Cq 9.13) to 10^{2} copies/µL (Cq 32.08). When a standard curve was plotted, the regression coefficient value was high (R^2 = 0.9950), and the efficiency of the reaction was calculated at 103.4541%, indicating that the reaction was optimized and reproducible (Fig. S4). The detection limit for EBOV was determined to be 10^{2} copies/µL. This detection limit is within similar and acceptable levels as other RT-qPCR assays.

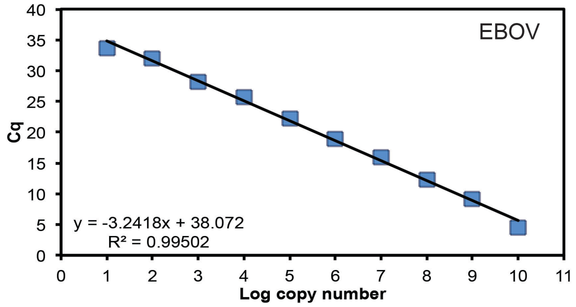

Figure S4. Limit of detection of the qPCR assay. Serial dilutions of 10^{10} copies to 10^{1} copies were assayed to determine the limit of detection for EBOV.
